# Effects of central nervous system electrical stimulation on non-neuronal cells

**DOI:** 10.3389/fnins.2022.967491

**Published:** 2022-09-15

**Authors:** Nathaniel P. Williams, Neetu Kushwah, Vaishnavi Dhawan, Xin Sally Zheng, Xinyan Tracy Cui

**Affiliations:** ^1^Department of Bioengineering, University of Pittsburgh, Pittsburgh, PA, United States; ^2^Center for the Neural Basis of Cognition, Pittsburgh, PA, United States; ^3^McGowan Institute for Regenerative Medicine, Pittsburgh, PA, United States

**Keywords:** electrical stimulation, non-neuronal cell types, microglia, astrocytes, oligodendrocytes, endothelial cells, blood brain barrier (BBB), neuroinflammation

## Abstract

Over the past few decades, much progress has been made in the clinical use of electrical stimulation of the central nervous system (CNS) to treat an ever-growing number of conditions from Parkinson’s disease (PD) to epilepsy as well as for sensory restoration and many other applications. However, little is known about the effects of microstimulation at the cellular level. Most of the existing research focuses on the effects of electrical stimulation on neurons. Other cells of the CNS such as microglia, astrocytes, oligodendrocytes, and vascular endothelial cells have been understudied in terms of their response to stimulation. The varied and critical functions of these cell types are now beginning to be better understood, and their vital roles in brain function in both health and disease are becoming better appreciated. To shed light on the importance of the way electrical stimulation as distinct from device implantation impacts non-neuronal cell types, this review will first summarize common stimulation modalities from the perspective of device design and stimulation parameters and how these different parameters have an impact on the physiological response. Following this, what is known about the responses of different cell types to different stimulation modalities will be summarized, drawing on findings from both clinical studies as well as clinically relevant animal models and *in vitro* systems.

## Introduction

Neurological disorders were the leading cause of disease burden globally as of 2015 according to the World Health Organization’s Global Burden of Diseases, Injuries and Risk Factors study (Feigin et al., 2017). In the United States alone, nearly 200 million people suffered from neurological disorders in 2017 (Collaborators et al., 2021) with an associated cost estimated at $765 billion or more, with the prevalence of neurological diseases in the United States on the rise from 1990 to 2017 (Gooch et al., 2017; Collaborators et al., 2021). Neuromodulation *via* electrical stimulation has become increasingly useful not only for the treatment of drug-resistant psychiatric disorders such as depression (Mayberg et al., 2005; McClure et al., 2015), but also for the treatment of neurological disorders including Parkinson’s disease (PD) (Deuschl et al., 2006), essential tremor and dystonia (Uc and Follett, 2007), epilepsy (Theodore and Fisher, 2007), and chronic pain (O’Connell et al., 2018). Its use has also enabled the partial restoration of sensory perception in patients suffering from loss of vision, hearing, and touch (Wilson and Dorman, 2008; Zeng et al., 2008; Micera et al., 2010; Ayton et al., 2014; Couloigner et al., 2014; Yue et al., 2015; Flesher et al., 2016, 2021; Schiefer et al., 2016; Sehic et al., 2016; Jang et al., 2019). This research has fueled the developing field of bioelectronic medicine, encompassing central nervous system (CNS) stimulation. The number of conditions for which electrical stimulation could become a viable treatment continues to increase. Studies of electrical stimulation of the autonomic and peripheral nervous system for the treatment of various conditions such as gastritis (Xu et al., 2017; Shine and Abell, 2020) and cardiovascular disease (He et al., 2016) as well as others (Clancy et al., 2014; Howland, 2014; Yang et al., 2018; Maisiyiti and Chen, 2019; Yap et al., 2020) have been explored. For the purposes of this review, we will narrow our focus to CNS stimulation, specifically, brain and spinal cord stimulation. Effects specific to electrical stimulation will be discussed, as distinct from the effects of device implantation, which have been reviewed elsewhere (Biran et al., 2005; Kozai et al., 2012, 2015; Saxena et al., 2013; Salatino et al., 2017; Woeppel et al., 2017; Bennett et al., 2018; Ereifej et al., 2018). Invasive stimulation modalities necessarily elicit a foreign body response to the implanted device, which will be discussed briefly as this is the context in which the response to electrical stimulation occurs. In characterizing the response to electrical stimulation *via* implanted devices, one of the challenges is to distinguish the response to stimulation from the response to device implantation (Deuschl et al., 2006; Cogan, 2008; Forst et al., 2015).

This review aims to provide readers in biology with information on electrode technologies and principles of electrical stimulation used in clinical modalities. For readers in material science this review aims to provide an overview of research investigating the effects of electrical stimulation of the CNS, with a focus on the effects on non-neuronal cell types. The effects of electrical stimulation on neurons have been extensively reviewed elsewhere (Ranck, 1975; Merrill et al., 2005; Liu et al., 2018).

## Clinically relevant approaches to electrical stimulation of the central nervous system

Clinically available electrical neuromodulation technologies have improved the quality of life of hundreds of thousands of patients living with neurological disorders, the most successful of which has been deep brain stimulation (DBS) for the treatment of movement disorders (Lozano et al., 2019). To achieve the desired therapeutic effects, the selection of electrode configuration and stimulation parameters is critical. The specificity of neuronal recruitment is governed by the placement, size, and material construction of the electrode device whereas the safety and efficacy of stimulation are largely influenced by electrode materials and stimulation paradigms. Electrode construction, material composition, and stimulation parameters for common clinical applications of electrical stimulation are summarized in Table 1 and described in the following sections. This does not represent an exhaustive list of paradigms as electrical stimulation is a rapidly evolving field, but rather this list touches on some of the more successful and more widely applicable clinical modalities in electrical stimulation of the central nervous system.

**TABLE 1:** Summary of stimulation parameters reported in literature for the major stimulation modalities discussed in this review.

Neuromodulation	Electrode type	Materials	Clinically approved stimulation paradigm	References
DBS	Macroelectrode	Pt/Ir	2–4 V, 60–450 μs pulse width, 130–185 Hz	Kuncel and Grill, 2004
	-	-	0.5–6.5 V, 60–180 μs pulse width, 100–185 Hz	Yang et al., 2020
SCS	Octopolar electrode	Pt/Ir	1–5 mA, 10 kHz for 30 μs	Kapural et al., 2015
	-	-	0.5 V, 120 μs pulse width, 40 Hz	Yuru Tang et al., 2018
RNS	Multicontact depth or strip electrodes	Pt/Ir	0.5–12.0 mA, 100 or 200 Hz, pulse width 160 μs, burst duration at 100 ms, charge density 6.1 μC/cm^2^	Jobst et al., 2017
	-	-	0.5–12 mA, pulse width 40–1,000 μs, 333 Hz	Matias et al., 2019
tDCS	Surface (scalp) electrode	Rubber	2 mA, 20 min.	Im et al., 2019; Khedr et al., 2019
ICMS	Bed-of-needles (Utah) microelectrode array	Sputtered IrO_2_ (SIROF)	14–64 μA, 200 μs cathodic pulse width, 100 μs interphase interval and 400 μs charge balanced anodic pulse width, 100 Hz	Flesher et al., 2021
	-	-	6–100 μA, 200 μs cathodic pulse width, 100 μs interphase interval and 400 μs charge balanced anodic pulse width, 25–300 Hz	Flesher et al., 2016
	-	-	20–100 μA, 200 μs cathodic pulse width, 53 μs interphase interval and 200 μs charge balanced anodic pulse width, 50–350 Hz	Salas et al., 2018

Depending on the treatment, electrical stimulation may be delivered invasively *via* macroelectrodes (geometric surface area > 5,000 μm^2^) (Deuschl et al., 2006) or microelectrodes (geometric surface area < 5,000 μm^2^) (Cogan, 2008) or non-invasively *via* surface electrodes (Forst et al., 2015). Current may be applied *via* alternating or direct current stimulation or *via* pulsatile stimulation. The vast design space of electrical stimulation, from electrode construction and material selection to stimulation paradigm, requires a deep understanding of the effects of electrical stimulation not only on neurons, but also on the non-neuronal cells of the nervous system.

### Invasive neurostimulation devices utilizing pulsatile current stimulation

We will first summarize a few of the more prominent neurostimulation modalities requiring surgical implantation into the central nervous system. These modalities for the most part employ alternating current stimulation, often utilizing biphasic pulsatile stimulation with pulse widths on the order of hundreds of microseconds. Target regions range from the cortex to subcortical ganglia to the spinal cord, and electrode configurations can be either macroelectrodes or microelectrodes depending on the therapeutic target. Of these modalities, DBS and ICMS have the largest literature and will be the focus of this review in subsequent sections. Other emerging modalities are mentioned here for completeness and to provide a wider overview.

#### Macrostimulation devices

##### Deep brain stimulation

Deep brain stimulation (DBS) is an invasive neuromodulation technique that involves a surgical procedure to implant a stimulating electrode to a targeted brain region with a subcutaneously implanted pulse generator (Amon and Alesch, 2017; Lozano et al., 2019). DBS has successfully treated and/or managed negative symptoms for patients with essential tremor (Nazzaro et al., 2013), Parkinson’s disease (Deuschl et al., 2006), dystonia (Miocinovic et al., 2013), obsessive disorder (Wu H. et al., 2021), epilepsy (Wu Y. C. et al., 2021), and chronic pain (Boccard et al., 2015). Globally, over 160,000 patients have undergone DBS treatment for several neurological conditions, with an increasing number of patients undergoing the procedure every year (Lozano and Lipsman, 2013). Clinical DBS leads have a variety of configurations, with one popular configuration having a diameter of 1.27 mm, a polyurethane outer jacket and four cylindrical or “ring shaped” electrode contacts. The conducting electrode surface is made of platinum-iridium (Pt/Ir) with a length of 1.5 mm and a surface area of ∼6 mm^2^ (Buhlmann et al., 2011). The large surface area typical of DBS electrodes puts them in the category of macroelectrodes as opposed to microelectrodes. The advantage of macroelectrodes is primarily in their ability to drive electrical changes to larger volumes of tissue (Butson and McIntyre, 2006). While this electrode design is less targeted, it is well-suited to generating a therapeutic effect in the subcortical nuclei of the basal ganglia which are the regions typically targeted by DBS.

##### Responsive neurostimulation

Responsive neurostimulation (RNS) is a brain-responsive neurostimulation system approved by the FDA to manage drug resistant seizures, which make up about 30–40% of the total number of patients with epilepsy. Nair et al. (2020) published a 9-year prospective study on the efficacy and safety of RNS for focal epilepsy and reported the median reduction in seizure frequency to be 75%. Stimulation was well-tolerated, and adverse events were similar to other neurostimulation devices. RNS is a closed-loop brain responsive neurostimulator. Unlike DBS, the RNS system includes a cranially implanted neurostimulator. Stimulator units can power both depth leads (1.27 mm diameter, 2 mm length, 0.08 cm^2^ surface area, Pt/Ir) and cortical strip leads (3.175 mm diameter, 0.08 cm^2^ surface area, Pt/Ir) depending on the epileptogenic loci being targeted (Jobst et al., 2017). Each lead typically contains four electrode contacts. *Via* these contacts, the electrocorticographic (ECoG) signal is continually monitored and the device is programmed by the physician to deliver stimulation in response to defined patient specific ECoG patterns which are determined by the physician to be predictive of a seizure. Stimulation parameters can also be adjusted by the physician to achieve the best seizure suppression. This closed-loop stimulation paradigm could have potential benefits compared to continuous stimulation, and it will be interesting to see how this technology develops.

##### Spinal cord stimulation

Spinal cord stimulation (SCS) is an invasive technique that can be used for the management of chronic pain. The SCS pulse generator is placed subcutaneously, and leads are placed in the spinal cord where electrical stimulation is delivered. However, as many as 30% of SCS patients fail to obtain long-term pain relief (De La Cruz et al., 2015). The electrode material used for spinal cord stimulation is the same as that used in DBS and RNS. A common lead construction is a paddle shaped lead with evenly spaced oval or circular electrode contacts in several rows, typically three or more, coming in close contact with the spinal cord once implanted, with contact sizes on the order of millimeters (Nitsche and Paulus, 2001; Fregni et al., 2005; Gandiga et al., 2006; Oakley et al., 2006; Boggio et al., 2007; Nitsche et al., 2008; DaSilva et al., 2011; Bennabi and Haffen, 2018; Milighetti et al., 2020; Solomons and Shanmugasundaram, 2020; Rigoard et al., 2021).

#### Microstimulation devices

##### Intracortical microstimulation

Intracortical microstimulation (ICMS) delivers small amounts of current through arrays of closely spaced microelectrodes implanted in the cortex, which enables the partial restoration of tactile sensation (Flesher et al., 2016, 2021; Schiefer et al., 2016; Salas et al., 2018) and visual perception (Murphey et al., 2009; Foroushani et al., 2018; Fernández et al., 2021). It is beneficial for achieving dexterous prosthesis control for humans with sensorimotor dysfunction (Fifer et al., 2020). ICMS employs microelectrodes with geometric surface areas typically less than 5,000 μm^2^ (Cogan et al., 2009; Wark et al., 2013; Pancrazio et al., 2017). The small electrode size enables high spatial selectivity which may allow for a wider range of stimulation parameters, evoking a wider range of perceived sensation in patients. The surface coating material for stimulating microelectrodes is typically a sputtered iridium oxide film, although other materials are also being tested, summarized by Zheng et al. (2021a) in a recent review.

### Non-invasive neurostimulation devices utilizing direct current stimulation

Although non-invasive neuromodulation of the CNS is being explored in a variety of paradigms, we will narrow our focus in this review to transcranial direct current stimulation (tDCS) as this is the most heavily studied. tDCS employs direct current stimulation and utilizes electrodes which are orders of magnitude larger than those used in implanted devices. One important consideration when it comes to non-invasive neurostimulation is the role of the intervening non-neuronal tissue.

#### Transcranial direct-current stimulation

Transcranial direct-current stimulation (tDCS) is a non-invasive form of electrical stimulation which delivers direct current of amplitudes in the range of 0.5–4 mA *via* electrodes placed on the scalp. While it has been shown in research to be effective for the treatment of depression (Bennabi and Haffen, 2018) and pain (O’Connell et al., 2018), it has not yet been approved as a treatment by the FDA. Direct current is delivered through scalp electrodes, which does not generate action potentials (Milighetti et al., 2020) but does lead to changes in excitability (Nitsche and Paulus, 2001). Anodal (positive) current has been shown to enhance motor cortex excitability, whereas cathodal (negative) current can reduce excitability (Nitsche et al., 2008). Important parameters include current densities, stimulation duration, electrode size, and electrode shape, which determine the safety of the brain stimulation. Most studies reported a current density between 0.029 and 0.08 mA/cm^2^, electrode size between 25 and 35 cm^2^ with a stimulation current of 1–3 mA for a duration of 20–30 min (Nitsche and Paulus, 2001; Fregni et al., 2005; Gandiga et al., 2006; Boggio et al., 2007; Solomons and Shanmugasundaram, 2020). Additionally, most studies utilized conductive rubber or metal electrodes that are embedded in a sodium chloride-soaked sponge (typically between 15 and 140 mM NaCl) (DaSilva et al., 2011). A comprehensive overview of the various tDCS stimulation protocols can be found elsewhere (Nitsche et al., 2008; Solomons and Shanmugasundaram, 2020).

Despite the clinical success of many electrical neurostimulation techniques, there are still challenges that require additional investigation. These challenges can be categorized into (1) charge injection limit, (2) material stability, and (3) tissue health and function. These issues are reviewed in detail by Merrill et al. (2005), Cogan (2008), and Zheng et al. (2021a). Investigation into the effects of electrical stimulation has been largely focused on neurons, with less emphasis on the roles that non-neuronal cells play. Understanding the response of non-neuronal cells to electrical stimulation will likely provide key insights into the overall biological response to electrical stimulation of the CNS, leading to more stable and effective interfaces for neural stimulation.

## Response of neurons and non-neuronal cell types to electrical stimulation

### Response of neurons to electrical stimulation

We will first discuss neurons as these are generally the primary cells of interest in studies of electrical stimulation. Although they are not the focus of this review, what is known about their response to electrical stimulation will provide context and background for understanding the response of non-neuronal cells to electrical stimulation.

#### Neurons

Neurons play the primary role in conducting electrical signals in the nervous system. Many studies have investigated the effect of various types of electrical stimulation on neurons, finding that the responses of neurons are highly modulated by the properties of the electrical field to which they are subjected (Florence et al., 2009; Aberra et al., 2018; Varoli et al., 2018; Jakobs et al., 2019). DBS and ICMS utilize pulsatile stimuli which are effective at eliciting action potentials by depolarizing neurons *via* the action of voltage-gated sodium channels (McIntyre and Anderson, 2016). The most likely compartment of neurons to be activated by electrical stimulation are the axon terminals due to the higher density and lower activation threshold of voltage-gated ion channels than those present in the soma (Aberra et al., 2018).

#### Deep brain stimulation

Under high-frequency (HF) DBS, neurons first exhibit altered firing patterns, with action potentials in axons becoming entrained to the stimulation frequency. DBS primarily affects axons, instead of dendrites or the cell bodies. This leads to both excitatory and inhibitory effects of DBS on neuronal activity. The net effect of these complex changes is that DBS leads to a depolarization block in neurons in the target region, preventing the regular firing of action potentials, however, due to the unstable nature of these interactions, periodic release from depolarization block and bursts of action potentials have been observed. All these factors in turn influence the behavior of the network to produce the therapeutic effect. These short-term electrophysiological effects are followed by alterations in neurotransmitter release and changes in the expression of various proteins such as c-Fos, VEGF, BDNF, GAP-43, synaptophysin and α-synuclein, some of which can take days or weeks to manifest (Delaville et al., 2011; Vedam-Mai et al., 2012; Jakobs et al., 2019).

#### Intracortical microstimulation

In ICMS, a smaller region of neurons around each electrode contact is stimulated to fire action potentials. Thus, the application of programmed timing and patterning of stimulation parameters to each electrode contact individually can produce a spatially and temporally resolved sensory percept (Parker et al., 2011). A study on the safety of chronic ICMS performed in feline cortex found that damage to neurons was related to the interaction between charge density and charge per phase. Tissue was stimulated at a range of parameters, from 800 to 1,600 μC cm^–2^, 52 to 104 nC ph^–1^, 130 to 260 μA for 7 h. They did not find evidence of damage to glia; however, they were primarily concerned only with neurons and used Nissl and H&E staining which could limit their ability to detect changes to glia (McCreery et al., 1990). Another study in non-human primates stimulated the cortex for 4 h per day, 5 days per week, for 6 months at amplitudes of 10–100 μA, with pulse train durations of 1 or 5 s, and duty cycles of 33–100%. Based on blinded grading of the post-mortem histology, they concluded that while there was considerable damage to the cortical tissue, there was no additional detectable effect from ICMS at the stimulation parameters used in their study. These results suggest that there may be an optimal range of stimulation parameters which can induce reliable activation of neurons and is low enough that it does not lead to excessive death or damage to neurons around the site of stimulation (Rajan et al., 2015). There are, however, limitations to these studies, such as having only a single timepoint of assessment and the nearby placement of electrodes in different conditions which may confound the results if the effects of stimulation are widespread, as well as the fact that gross morphology and/or total cell count were evaluated, rather than the distribution and structure of cells surrounding stimulated electrodes. Therefore, further work on understanding the response of neurons to electrical stimulation should be conducted using new technologies such as multiphoton imaging techniques to image the response of live brain tissue in real time at cellular resolution. These techniques have been refined and their utilization provides insights into the effects of electrode implantation on many aspects of the tissue response such as microglia morphology and behavior, neuronal morphology and activity, and changes to the vasculature and the blood brain barrier (BBB) (Kozai et al., 2012, 2016; Eles et al., 2018, 2019; Michelson et al., 2018; Yang et al., 2021; Zheng et al., 2021b). These techniques have been adapted to study changes induced by electrical stimulation that are separate from those induced by the implantation of the electrode itself (Zheng et al., 2021b). More studies along these lines are needed to develop a complete picture of the way electrical stimulation influences the different cell types of the brain over time *in vivo* under different stimulation parameters.

#### Transcranial direct current stimulation

The effects of tDCS on neurons have been investigated *via* both experimental and modeling studies (Denoyer et al., 2020). tDCS does not directly trigger action potentials but modulates neuronal excitability by shifting the resting membrane potential (Milighetti et al., 2020). In general, anodal tDCS is found to be excitatory while cathodic is found to be inhibitory, although this may vary by brain region (Varoli et al., 2018). At the cellular level, the stimulation effects depend on cell morphology and the electrical field direction (Chung et al., 2020). In mice, anodal DCS has been reported to increase spine density and induced structural synaptic plasticity in the cortex when paired with an external stimulus in a BDNF dependent manner (Gellner et al., 2020). A reduction in the number of neurons in the stimulated area of cortex was seen in mice stimulated with tDCS at a high charge density of 198 kC/m, accompanied by an increase in neurogenesis in the subventricular zone based on DCX staining (Pikhovych et al., 2016). tDCS also led to an increase in mRNA levels of proteins that play a role in synaptogenesis and axonal development and regeneration such as BDNF, Synapsin I, calcium/calmodulin-dependent protein kinase type II (CaMKII), CREB and c-FOS as measured by RT-qPCR analysis in rats stimulated daily for 7 days. In an *in vitro* hippocampal slice study, applying direct current during plasticity induction boosted the extent of long-term potentiation (LTP) (Kronberg et al., 2020). It also triggered alterations in postsynaptic membrane potential during endogenous synaptic activity, although it’s unclear how comparable these findings are to stimulation conditions used in human patients.

### Response of non-neuronal cells to electrical stimulation

This review focuses primarily on what is known of the effects of different electrical stimulation paradigms on non-neuronal cell types. While some forms of non-invasive neural stimulation are employed, invasive neuromodulation necessarily requires the implantation of an electrode device into the brain which triggers a cascade of foreign body responses that activate microglia and astrocytes, while damaging oligodendrocytes and the integrity of the BBB (Salatino et al., 2017). While not the focus of this review, it is important to understand the effects electrode implantation has on neural tissue to understand the importance of differentiating these changes from those induced by electrical stimulation. We will briefly discuss what is known of the response of cells upon device implantation as this is the context in which the response to electrical stimulation occurs. Upon device implantation, microglia are immediately activated and produce a variety of inflammatory molecules which signal monocytes and astrocytes, recruiting them to the site of the implant. Activated microglia and blood-derived macrophages persist at the periphery of implants for the duration of implantation and are surrounded by a dense accumulation of astrocytes, frequently referred to as the glial scar (Woeppel et al., 2017). Responses of different non-neuronal cell types to different prominent stimulation modalities discussed in this review are summarized in Table 2.

**TABLE 2:** Summary of the major effects of electrical stimulation on non-neuronal cells for the modalities addressed.

Cell type	Function	ES modality	Morphological effects	Biochemical/Molecular effects	References
Microglia	*Native immune cells of the central nervous system	DBS	*Transition to amoeboid morphology *Increased phagocytosis *Hypertrophy	Alterations in neurotransmitter release, protein expression, and receptor dynamics after DBS can have network and biochemical effects on microglia *via* neuron-microglia crosstalk	Vedam-Mai et al., 2016; Jakobs et al., 2019
		tDCS	*Align parallel to the electrical field *Increased ramified morphology, motility, and increased phagocytic function *Hypertrophy and decreased surveillance *Cathodal stimulation supports M1-polarization *Increased markers of inflammation such as TNF-alpha and interleukins	*Increased BDNF expression, promoting synaptic plasticity *Increased activation of pro-inflammatory and anti-inflammatory phenotypes *Increased expression of COX-2, iNOS and PGE2	Braun et al., 2016; Gellner et al., 2016, 2021; Mishima et al., 2019; Korai et al., 2021
		ICMS	*Decreased proliferation	*Increased trophic factors	Baba et al., 2009
Astrocytes	*Axon guidance and synaptic support *Control of BBB and blood flow	DBS	*Activation *Reactive gliosis *Hypertrophy *Release of neurotransmitters and neuromodulators *Increased hypermorphic reactive astrocytes thought to alter neuronal signaling *Astrocyte mediated alteration of cerebral blood flow	*Enhanced intracellular Ca2 + and release of gliotransmitters *Release of extra−synaptic glutamate which triggers local Ca2 + response facilitated through mGluR5 *Downregulation of astrocytic metabolism while promoting the secretion of matricellular proteins *Release of glutamate and ATP, both of which modify neuronal networks	Vedam-Mai et al., 2012; Fenoy et al., 2014
		tDCS	*Protrusion elongation appears at low field strength *Align perpendicular to the electrical field at high field strength	*Increased Ca^2+^ which promotes cortical plasticity *Increased synaptic transmission *via* increased intracellular Ca^2+^	Pelletier et al., 2014; Monai et al., 2016; Mishima et al., 2019
		ICMS	*Increased activation in a central pain syndrome model *Decreased activation in an ischemic stroke model	*Increased trophic factors	Baba et al., 2009; Cha et al., 2020
Oligo-dendrocytes	*Myelin production *Metabolic support to myelinated axons	DBS	*High frequency stimulation leads to increased proliferation and differentiation of oligodendrocyte precursor cells	-	Nagy et al., 2017
		tDCS	*Cathodal stimulation recruits oligodendrocyte precursors to the lesion site in focal cerebral ischemia and increases the proliferation of oligodendrocyte precursor cells	-	Braun et al., 2016
		ICMS	*Increased oligodendrocyte differentiation, maturation, and myelination	-	Lee et al., 2017

#### Microglia and astrocytes

Microglia are the resident immune cells of the CNS (Kettenmann et al., 2011) and exist in the healthy CNS in an inactive and ramified state with a round cell body and extensive processes that survey their surrounding environment constantly (Nimmerjahn et al., 2005). Upon injury of the CNS, microglia are the first cells to respond and adopt an activated amoeboid morphology, followed by cell proliferation, migration, and encapsulation of the damage site (Sofroniew and Vinters, 2010; Kozai et al., 2012). Biran et al. (2005) showed that chronic implantation significantly increased the density of activated microglia as early as 24 h after implantation and that the high microglia density continued for the entire implantation period. Kozai et al. (2012) used *in vivo* two-photon microscopy to study microglia behavior in real-time during and immediately after the implantation of a neural probe. Upon implantation, microglial cells directly adjacent to the inserted probes extended their processes toward the probe at a rate of (1.6 ± 1.3) μm min^–1^ for 30–45 min without significant cell body movement over the first 6 h after the probe implantation. Six hours after probe implantation, 50% of the microglia within 130.0 μm of the probe surface showed morphological characteristics of transitional stage (T-stage) activation, generating limited new processes that rapidly extended while most were withdrawn (Kozai et al., 2012; Robin et al., 2018; Borrachero-Conejo et al., 2019; Chen et al., 2021).

Astrocytes are a subtype of glial cells within the CNS. They have a star-shaped morphology, and their processes surround both neuronal synapses and blood vessels. They play a key role in the homeostasis of glutamate, various ions such as Ca^2+^, K^+^, and water, and defend against oxidative stress, assist in scar formation, and tissue repair (Sofroniew and Vinters, 2010). They have also been shown to have a critical role in inhibitory signal transmission (Robin et al., 2018; Chen et al., 2021). In a study of electrical stimulation by organic cell-stimulating and sensing transistors, stimulation elicited an intracellular Ca^2+^ increase in astrocytes, hinting at a more active response to electrical stimulation (Borrachero-Conejo et al., 2019). Astrocytes are also involved in the response to injury. Following the initial microglia response, they are recruited to the site of implantation and respond by increasing in size and an upregulation of GFAP intermediate filament protein, a hallmark of astrocyte activation, as well as releasing factors which contribute to promoting the ongoing foreign body response. They eventually form a dense layer of cells which surround the device by typically 4–6 weeks post implant and create a barrier between electrode sites and surrounding neurons (Vedam-Mai et al., 2011; Kozai et al., 2015; Malaga et al., 2015; Salatino et al., 2017).

##### Deep brain stimulation

The therapeutic effect of DBS is likely influenced by several mechanisms including local and network-level electrical and neurochemical changes, modulation of oscillatory activity, synaptic plasticity, neuroprotection, and neurogenesis (Herrington et al., 2016). Due to the growing recognition of the importance of microglia and astrocytes in these processes, studying their responses to DBS is becoming an active area of investigation.

Vedam-Mai et al. (2016) performed a comparative study on the tissue response to DBS and demonstrated a reciprocal relationship of microglia and neural precursor cells in the presence of acute high-frequency stimulation. Specifically, they found a significantly higher density of ameboid microglia in the STN of rats who did not receive DBS (no stimulation or lesion only) compared to those that received DBS. Meanwhile, there was a significant increase in neural progenitor cells in the stimulated animals (Vedam-Mai et al., 2016). In addition, HF-DBS might also lead to an increase in the proliferation of neurogenic astrocytes, which can differentiate into neurons (Vedam-Mai et al., 2012). These findings suggest that electrical stimulation in the context of DBS may have an anti-inflammatory effect on microglia and lead to an increase in the proliferation of cells around the stimulating electrodes. These findings are corroborated by observations in human postmortem PD tissue which demonstrated reduced activation of microglia in the STN of high frequency DBS cases compared to cases in which DBS was not received. These observations suggest a potential reduction in neuroinflammation as a result of electrical stimulation of the STN. Another group found a significant upregulation of an angiogenic factor, vascular endothelial growth factor (VEGF), and downregulation of inflammatory processes in STN-DBS PD patients compared to non-DBS PD patients (Pienaar et al., 2015). This evidence suggests that microglia may play a critical role in the mechanisms underlying the therapeutic effects of DBS.

**FIGURE 1:**
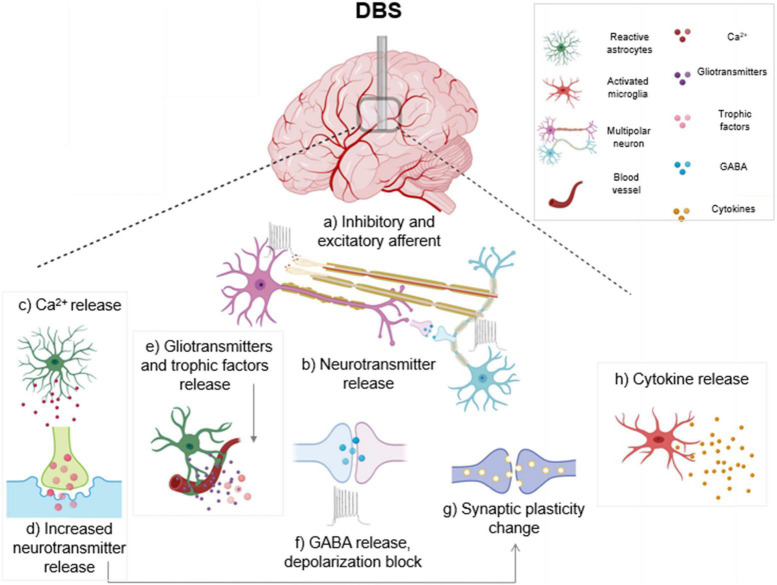
Electrical and cellular effects of deep brain stimulation (DBS): High frequency DBS causes axonal action potentials and depolarization of inhibitory and excitatory fibers projecting to target neurons (a) and induces release of neurotransmitters (b). In response to stimulation, activated astrocytes release calcium (c), gliotransmitters and trophic factors (e). Increased calcium from activated astrocytes also triggers neurotransmitter and gliotransmitter release (d) which can modulate synaptic transmission. DBS causes subsequent release of the inhibitory transmitter γ-aminobutyric acid (GABA), and a depolarization block which causes reduced activity in neuronal cell bodies (f). Stimulation can also generate synaptic plasticity changes, which can lead to long term potentiation (g). DBS induces an activated ameboid morphology in microglia and an upregulation of cytokines is observed (h). Boxed panels indicate effects on non-neuronal cell types (Hamani and Temel, 2012; Jakobs et al., 2019; Lozano et al., 2019).

*In vitro* high frequency stimulation of cultured astrocytes from PD model rats, using stimulation parameters that mimicked *in vivo* DBS, resulted in decreased levels of NF-κB in the cultured astrocytes. Additionally, high frequency stimulation inhibited IκB-α (an NF-κB cytoplasmic inhibitor protein) deterioration and hindered TNF-α-prompted NF-κB activation and p65 nuclear translocation, supporting an anti-inflammatory role for high frequency stimulation (HFS) in astrocytes in a PD rat model (Campos et al., 2020). Therefore, high frequency DBS potentially acts as a barrier to neuroinflammation by managing NF-κB levels through a direct effect on astrocytes. Figure 1 illustrates the various impacts of DBS, including inhibitory and excitatory effects on neuronal populations along with changes in astrocyte and microglia activation.

##### Intracortical microstimulation

In ICMS, small electrical currents are delivered into the intracortical space of the brain. The delivery of ICMS requires the implantation of an electrode device into the cortex, which triggers the foreign body response and induces microglial activation. An increasing number of studies are investigating the effect of electrical stimulation of the cortex on microglia and overall brain health. Baba et al. (2009) characterized the effect of electrical stimulation on cerebral ischemia and found neuroprotective properties of electrical stimulation in a rat model of acute stroke. Specifically, they found that cortical stimulation prevented the ischemia-associated increase in apoptotic cells in the injured cortex by activating anti-apoptotic cascades through the phosphoinositide 3-kinase pathway compared to the uninjured cortex. As a result, electrical stimulation of the ischemic brain promoted angiogenesis and reduced microglia proliferation, resulting in reduced infarct volumes, and improved functional recovery (Baba et al., 2009). In a study of central pain syndrome in a rat model, electrical stimulation in the motor cortex modulated the activity of astrocytes in zona incerta, which they found to be decreased in central pain syndrome rats but recovered to control levels with motor cortex stimulation. This suggests that electrical stimulation of glia could have far reaching effects projecting to brain areas outside the stimulated region (Cha et al., 2020).

##### Transcranial direct current stimulation

Transcranial direct current stimulation delivers a relatively weak electrical field to a large volume of tissue through non-invasive scalp electrodes, which is thought to induce changes in neural excitability. *In vitro* studies of glia exposed to direct current electric fields show that in response to higher intensity fields, astrocytes align perpendicularly to the applied electric field, while microglia generally respond to the electric fields by changing morphology or expression of neuroinflammatory markers. For example, increased pro-inflammatory COX-2 production was found in microglia exposed to a 100 mV/mm electric field (Pelletier et al., 2014). The main limitation of relating direct current stimulation experiments performed in cell culture to tDCS paradigms used in humans is that the stimulation parameters do not align well. In this study the maximum field strength used of 100 mV/mm is approximately 100 times higher than what is used in humans, and this is typical of many *in vitro* studies of direct current stimulation. *In vitro* assays also generally do not consider that the applied voltage will be much attenuated after passing through the scalp and skull. *In vivo* direct current stimulation in mouse sensory cortex indicated that tDCS directly affects astrocytes as indicated by an almost immediate increase in astrocytic Ca^2+^, which could be a driver of tDCS-mediated LTP (Monai et al., 2016). In this *in vivo* study, like in many tissue culture studies, the electric field strength was over 50 times higher than that typically used in humans. Voltage sensitive transporters, ion channels and gap junction coupling are a few of the important factors implicated in the observed voltage-dependent effects of DCS on astrocytes, however it is unclear to what extent these factors are present at the relatively lower current densities used in humans (Gellner et al., 2016). Future *in vitro* and *in vivo* studies must attempt to employ electrical field parameters that more closely mimic what brain tissue is experiencing under stimulation parameters used in humans in order to more accurately inform the therapeutic efficacy of tDCS.

In an ischemic stroke mouse model, cathodal tDCS was delivered (55.0 A/m^2^) 30 min after middle cerebral artery occlusion. Subsequently, an acute decrease in Iba1 and CD45 positive cells in the injured region was observed compared to sham treated animals (Peruzzotti-Jametti et al., 2013). Iba1 expression is associated with microglial activation in the ischemic brain (Ito et al., 2001). Another study investigated the effects of cathodal and anodal tDCS and discovered an early upregulation of Iba1 positive activated microglia in both groups. However, after 10 days of cathodal tDCS, a 60% increase in the endogenous proliferating neural stem cells was observed, alluding to the capability of cathodal tDCS to mobilize stem cells and prompt their multiplication, thereby potentially conferring a neuroprotective effect as a result of stem cell mobilization (Rueger et al., 2012). This effect was absent in the anodal and sham tDCS groups, indicating the potential benefits specific to cathodal tDCS for the treatment of stroke patients. Another study investigated the effects of anodal tDCS on microglial morphology and mobility in awake mice and discovered increased soma enlargement as well as a reduction in microglial motility. They also showed the influence of noradrenaline in tDCS by depleting noradrenergic cells in the locus coeruleus using the selective toxin DSP4. In these mice, there was no effect of tDCS on the cell morphology of microglia (Mishima et al., 2019). For a detailed review of the effects of tDCS on cellular and inflammatory pathways, see Pelletier and Cicchetti (2014).

#### Oligodendrocytes

Oligodendrocytes are a type of glial cell that are produced from oligodendrocyte precursor cells (OPCs). One of their significant roles is to provide axons with insulation for efficient signal transduction. Myelinating oligodendrocytes enwrap neuronal axons with myelin, providing electrical insulation which can dramatically increase the speed of nerve impulse propagation (Bradl and Lassmann, 2010). One study investigated the effect of stimulating the corpus collosum through 25 μm platinum wire microelectrodes at different stimulation frequencies on the subsequent proliferation and differentiation of oligodendrocytes. They found that stimulation at 5 Hz was more effective in promoting differentiation of OPCs into oligodendrocytes, while stimulation at 25 Hz led to increased proliferation of OPCs. Based on caspase staining, their stimulation paradigm did not induce cell death in the corpus callosum. In the same study, using slice electrophysiology, they also found that stimulation frequency influenced the quantity and timing of glutamate release at the neuron-OPC synapse, suggesting that OPCs may respond differentially to different firing patterns of neurons and by this mechanism myelination may be tuned (Nagy et al., 2017).

In a rat stroke model, tDCS resulted in accelerated functional recovery *via* multiple cellular mechanisms including the recruitment of OPCs. The study indicated that both anodal and cathodal tDCS accelerated functional recovery and that following cathodal tDCS delivery, the oligodendrocyte precursors were observed to migrate toward the injury site, while microglia underwent M1 polarization. Their results corroborate the findings of Miron et al. (2013), which showed that M1-macrophages dominate early after demyelination and support proliferation and migration of oligodendrocyte precursors, while a later M2-polarization can induce the terminal differentiation of the precursors into mature oligodendrocytes (Braun et al., 2016). Studying the effects of several types of stimulation on migration and proliferation of oligodendrocytes and their relationship with other glial types as well as neurons is an active field of research, which can better inform the delivery of stimulation parameters to drive desired functional outcomes. For an in-depth review of the role of OPCs and oligodendrocytes in central nervous system insult, see Wellman et al. (2018).

#### Endothelial cells and the blood brain barrier

Endothelial cells (ECs) line the blood vessels and in the brain, ECs form tight junctions, and along with basement membrane, glial membrane, and projections of astrocytes make up the blood brain barrier. The BBB is the barrier between the cerebral capillary blood and the interstitial fluid of the brain (Wolburg et al., 2009). The vascular blood-brain barrier is comprised primarily of highly specialized brain endothelial cells (BECs) which modulate the flow of substances into and out of the brain. The BBB modulates neuroimmune communication through several complex pathways, which can be categorized as (1) modulation of BBB permeability, (2) immune regulation of BBB transporters and release of signaling molecules, (3) immune cell trafficking. These specialized brain endothelial cells also help to regulate the functions of other resident brain cells such as astrocytes, neurons, microglia, and oligodendrocytes (Erickson and Banks, 2018). BBB disruption is implicated in central nervous system disorders such as epilepsy, multiple sclerosis, PD, and Alzheimer’s disease (Farkas and Luiten, 2001; van Vliet et al., 2007; Pienaar et al., 2015; Erickson and Banks, 2018), as well as others. Understanding its function and dysregulation upon insult is of particular importance for understanding the response to brain interfacing devices, as invasive neural implants necessarily involve disruption of the BBB. It is thus critical to study the effect of therapeutic stimulation on the BBB and brain endothelial cells to understand how their function is affected and may help improve recovery upon device implantation or in specific disease states.

While the focus of this review is on the response to electrical stimulation as distinct from device implantation, we will summarize key findings related to the effects of device implantation, traumatic injury and disease as they relate to endothelial cells and the BBB here in order to highlight the importance and difficulty of disentangling these effects from the response to electrical stimulation when it comes to brain implanted devices. Specifically, it has been shown that the implantation of DBS electrodes does induce the mechanical breakdown of the BBB, which can intensify the inflammatory response and negate some of the benefits from stimulation (Kim et al., 2013). These difficulties are particular to invasive stimulating devices as in these cases stimulation will be acting on tissue injured by the device implantation itself, whereas non-invasive stimulation (e.g., tDCS) will be acting on uninjured tissue, although often under the influence of a disease state and not in a healthy brain.

##### Deep brain stimulation

Several studies investigating the involvement of BBB disruption in epileptic seizures have demonstrated that BBB breakdown may induce seizures and, conversely, seizure-induced BBB breakdown may cause further epileptic episodes (van Vliet et al., 2007). Albumin extravasation is one of the effects of increased BBB permeability, which can trigger neuroinflammation (Cacheaux et al., 2009; Ralay Ranaivo and Wainwright, 2010) and is implicated in disorders such as epilepsy. Deep brain stimulation of the anterior nucleus of the thalamus, which is involved in the spread of localized seizures, was shown to reduce albumin extravasation in epileptic rodents (Hamani and Temel, 2012; Kim et al., 2013; Chen et al., 2017; Jakobs et al., 2019; Lozano et al., 2019).

##### Intracortical microstimulation

Changes induced in the BBB and endothelial cells have mostly been studied in the context of implantation trauma caused by the mechanical insertion of microelectrodes and the subsequent effect of this BBB breach on device function (Saxena et al., 2013). We will briefly summarize these effects here before delving into what is known regarding electrical stimulation in this context. Acutely, device implantation causes a physical breach in the BBB which prompts the release of neurotoxic serum proteins such as albumin and fibronectin, pro-inflammatory cells, and cytokines that lead to neurodegeneration and cell death. Additionally, the release of these blood-serum proteins provokes the activation of the adjacent microglia and astrocytes (Salatino et al., 2017). One study found that the gene expression levels for tight junction and adherent junction proteins were reduced after microelectrode implantation and linked to persistent BBB breakdown (Bennett et al., 2018). In the chronic stage, further neuron loss and an increase in activated microglia and astrocytes takes place in the surrounding tissue, leading to gliosis. Additionally, cytokines and reactive oxygen and nitrogen species (ROS and RNS) released by microglia lead to neuroinflammation that decreases electrode performance. Intracortical microelectrode implantation prompts the overproduction of ROS and encourages neuronal impairment (Ereifej et al., 2018).

Regarding microstimulation, there is a shortage of studies investigating the mechanisms through which electrical stimulation can directly induce changes in the BBB and endothelial cells. One feline study reported evidence that electrical stimulation of the cortex induced BBB permeability changes above a charge injection threshold of 0.45 μC/phase for prolonged periods (36 h of stimulation), but that the BBB damage appeared to heal 1 month after stimulation (Pudenz et al., 1975). This suggests that microstimulation can indeed affect BBB permeability, although the exact mechanisms are not yet fully understood. Another study, which employed bipolar stimulation using coaxial electrodes placed in the locus coeruleus, found that stimulation induced an increase in BBB permeability, which was likely due to the release of noradrenaline as it was blocked by the administration of adrenergic receptor blockers (Sarmento et al., 1994). This further suggests that changes to the BBB induced by microstimulation may vary with the brain regions being stimulated, and the neurotransmitters involved. The paucity of data on the subject highlights the need for further research in this area (Saxena et al., 2013; Salatino et al., 2017; Bennett et al., 2018; Ereifej et al., 2018).

##### Transcranial direct current stimulation

**FIGURE 2:**
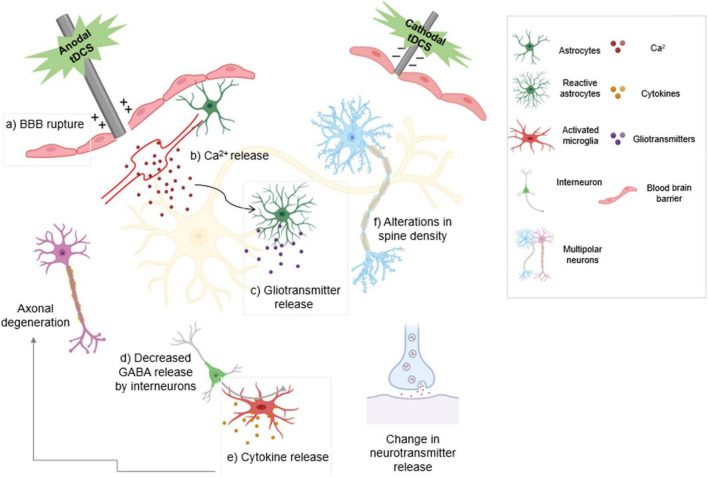
The type of stimulation determines the modulatory effect of transcranial direct current stimulation (tDCS). Anodal stimulation depolarizes the neuronal membrane and enhances neuronal excitability. Cathodal stimulation hyperpolarizes the neuronal membrane and reduces neuronal excitability. Anodal tDCS exacerbates bleeding and damage to the blood-brain barrier following ischemic injury. (a) tDCS activates noradrenergic fibers that release noradrenaline, increasing (b) astrocytic Ca^2+^ levels and release of gliotransmitters (c). Decreased GABA release by both anodal and cathodal tDCS results in decreased inhibition (d). Activated microglia release cytokines that cause inflammation and result in axonal degeneration (e). Anodal tDCS is involved in increasing dendritic spine density (f). tDCS also leads to change in neuronal neurotransmitter release. Boxed panels indicate effects on non-neuronal cell types.

Release of VEGFs and nitric oxide have been demonstrated to be the direct impacts of electrical stimulation on endothelial cells (Zhao et al., 2004; Peruzzotti-Jametti et al., 2013). One study indicated that DCS can regulate water permeability in an *in vitro* BBB model *via* electroosmosis (Cancel et al., 2018). DCS of cultured endothelial cells with a current range and duration of 0.1–1 mA and 20 min significantly and transiently increased the solute permeability of the BBB. Anodal tDCS can intensify cortical hemorrhage and provoke injury to the BBB when used to treat ischemia (Peruzzotti-Jametti et al., 2013). Secretion of noradrenaline increases astrocytic Ca^2+^ and gliotransmitter release. Both anodal and cathodal tDCS cause a decrease in GABA release, resulting in a reduction in inhibition. tDCS increases the number of activated microglia which secrete cytokines that cause neuroinflammation, although under certain conditions it has been shown to have an anti-inflammatory effect (Figure 2; Peruzzotti-Jametti et al., 2013; Pelletier and Cicchetti, 2014; Monai and Hirase, 2018; Mishima et al., 2019).

#### Electrical stimulation induced molecular changes

Electrical stimulation has been shown to modulate cellular activity by changing downstream signaling pathways such as MAPK-ERK1/2 and PI3K/Akt which are responsible for cell migration, survival, and growth (Chen et al., 2019). Furthermore, chronic electrical stimulation also leads to an elevation in Janus kinase-signal transducer and activator of transcription (JAK/STAT) signaling, which is critical for cytokine/chemokine signaling (Zareen et al., 2018). Another study demonstrated increased c-Fos in brain regions including cingulate cortex, posterior hypothalamus, and subiculum following DBS (Gimenes et al., 2019). Microstimulation of sensorimotor cortex also leads to the expression of c-Fos and c-Jun in the striatum (Parthasarathy and Graybiel, 1997). Like DBS and microstimulation, continuous A-tDCS exposure in the cortex also increased c-Fos mRNA levels (Kim et al., 2017). STN-HFS for 3 h in a PD rodent model has been shown to modulate the expression of the tyrosine hydroxylase gene, a key enzyme in dopamine synthesis. The therapeutic effects of HFS–DBS are also supported by a downregulation of CaMKIIa and Homer1, a protein associated with glutamate neurotransmission. This downregulation could bring about decreased sensitivity to glutamate in the basal ganglia downstream of the STN (Henning et al., 2007). Another study also indicated an increase in the expression of various trophic factors such as BDNF and VEGF and the synaptic markers GAP-43, synaptophysin, and α-synuclein in the fornix following acute DBS. Interestingly, this change occurred within 2.5 h after the stimulation began, and returned to baseline levels after 5 h (Gondard et al., 2015). An A-tDCS study also reported increased mRNA expression of genes associated with synaptic plasticity such as BDNF, CREB, Synapsin I, and CaMKII following continuous stimulation (Kim et al., 2017). In an analysis of a rat model of electrical modulation therapy in spinal cord stimulation for the treatment of neuropathic pain, the authors found that anodal SCS decreased the expression of various glial-specific genes associated with inflammatory responses and microglial activation (Itgb2, Cd74, Cd68) and reactive astrocytes (Cxcl16, Tlr2) (Vallejo et al., 2020; Cedeño et al., 2021). In a study investigating SCS in a porcine model of ischemia-reperfusion triggered ventricular arrhythmia as a treatment for sudden cardiac death, the authors found that cardiac ischemia increased c-Fos expression in the spinal cord, specifically in microglia and astrocytes. Furthermore, they found that SCS treatment, which was effective at reducing cardiac arrhythmia, was accompanied by a reduction in c-Fos expression specifically in microglia and astrocytes and an increase in c-Fos expression in inhibitory interneurons in the deep dorsal laminae of the dorsal horn of the spinal cord (Howard-Quijano et al., 2021). These studies suggest that, at least in the spinal cord, electrical stimulation appears to have an anti-inflammatory and neuroprotective effect that is mediated by microglia and astrocytes. Further study into the mechanisms of these effects, as well as the effects of electrical stimulation on microglia and astrocytes in the cortex, are needed to fully understand the potential benefits this may have for devising future therapies.

## Conclusion and future directions

Electrical stimulation of the CNS has been impactful not only on the treatment of neurological disorders, but also in the exploration of the neural circuitry involved in their pathologies as well as in the normal functioning of the healthy brain. The delivery of electrical current to the brain can cause direct or indirect excitation and inhibition of neuronal firing as well as induce changes in non-neuronal cell types. This review provides a survey of common clinically relevant stimulation approaches, focusing on what is known about their effect on non-neuronal cells including microglia, astrocytes, oligodendrocytes, and endothelial cells.

In the past few decades, there has been significant advancement in the field of electrical stimulation, and many studies have investigated the efficacy of electrical stimulation as a treatment for many neurological disorders. Electrical stimulation is only recently beginning to be fully appreciated for its applications in medicine. Beyond neurological conditions clinicians are beginning to explore how it can be applied in other ways such as potential treatments for cancer and genetic disorders in the nascent but booming field of bioelectronic medicine. Additional studies have focused on improving the stimulation efficiency and durability of implantable probes as well as the integration of different technologies into multifunctional probes for recording, stimulation, electrochemical sensing, and optogenetics. However, there is still much work to be done on these fronts. There are many underlying mechanisms governing the responses of many types of cells in the brain to electrical signals which are only now beginning to be understood. With further studies, many of these mechanisms could be harnessed, potentially providing effective treatments for a host of disorders, neurological and otherwise.

## Author contributions

NK wrote the first draft of the manuscript. NW made significant critical revisions and wrote sections of the manuscript. VD and XSZ wrote sections of the manuscript. XC defined the topic and scope of the review and supervised the writing efforts. All authors contributed to manuscript revision and read and approved the submitted version.

## References

[B1] Aberra A. S. Peterchev A. V. Grill W. M. (2018). Biophysically realistic neuron models for simulation of cortical stimulation. *J. Neural. Eng.* 15:066023. 10.1088/1741-2552/aadbb1 30127100 PMC6239949

[B2] Amon A. Alesch F. (2017). Systems for deep brain stimulation: review of technical features. *J. Neural. Transm.* 124 1083–1091. 10.1007/s00702-017-1751-6 28707160 PMC5565669

[B3] Ayton L. N. Blamey P. J. Guymer R. H. Luu C. D. Nayagam D. A. Sinclair N. C. (2014). Australia Research, First-in-human trial of a novel suprachoroidal retinal prosthesis. *PLoS One* 9:e115239. 10.1371/journal.pone.0115239 25521292 PMC4270734

[B4] Baba T. Kameda M. Yasuhara T. Morimoto T. Kondo A. Shingo T. (2009). Electrical stimulation of the cerebral cortex exerts antiapoptotic, angiogenic, and anti-inflammatory effects in ischemic stroke rats through phosphoinositide 3-kinase/Akt signaling pathway. *Stroke* 40:e598–e605. 10.1161/STROKEAHA.109.563627 19762690

[B5] Bennabi D. Haffen E. (2018). Transcranial Direct Current Stimulation (tDCS): A Promising Treatment for Major Depressive Disorder? *Brain Sci.* 8:81. 10.3390/brainsci8050081 29734768 PMC5977072

[B6] Bennett C. Samikkannu M. Mohammed F. Dietrich W. D. Rajguru S. M. Prasad A. (2018). Blood brain barrier (BBB)-disruption in intracortical silicon microelectrode implants. *Biomaterials* 164 1–10. 10.1016/j.biomaterials.2018.02.036 29477707 PMC5895107

[B7] Biran R. Martin D. C. Tresco P. A. (2005). Neuronal cell loss accompanies the brain tissue response to chronically implanted silicon microelectrode arrays. *Exp. Neurol.* 195 115–126. 10.1016/j.expneurol.2005.04.020 16045910

[B8] Boccard S. G. Pereira E. A. Aziz T. Z. (2015). Deep brain stimulation for chronic pain. *J. Clin. Neurosci.* 22 1537–1543. 10.1016/j.jocn.2015.04.005 26122383

[B9] Boggio P. S. Nunes A. Rigonatti S. P. Nitsche M. A. Pascual-Leone A. Fregni F. (2007). Repeated sessions of noninvasive brain DC stimulation is associated with motor function improvement in stroke patients. *Restor. Neurol. Neurosci.* 25 123–129. 17726271

[B10] Borrachero-Conejo A. I. Saracino E. Natali M. Prescimone F. Karges S. Bonetti S. (2019). Electrical Stimulation by an Organic Transistor Architecture Induces Calcium Signaling in Nonexcitable Brain Cells. *Adv. Healthc. Mater* 8:e1801139. 10.1002/adhm.201801139 30565894

[B11] Bradl M. Lassmann H. (2010). Oligodendrocytes: Biology and pathology. *Acta Neuropathol.* 119 37–53. 10.1007/s00401-009-0601-5 19847447 PMC2799635

[B12] Braun R. Klein R. Walter H. L. Ohren M. Freudenmacher L. Getachew K. (2016). Transcranial direct current stimulation accelerates recovery of function, induces neurogenesis and recruits oligodendrocyte precursors in a rat model of stroke. *Exp. Neurol.* 279 127–136. 10.1016/j.expneurol.2016.02.018 26923911

[B13] Buhlmann J. Hofmann L. Tass P. A. Hauptmann C. (2011). Modeling of a segmented electrode for desynchronizing deep brain stimulation. *Front. Neuroeng.* 4:15. 10.3389/fneng.2011.00015 22163220 PMC3233722

[B14] Butson C. R. McIntyre C. C. (2006). Role of electrode design on the volume of tissue activated during deep brain stimulation. *J. Neural. Eng.* 3 1–8. 10.1088/1741-2560/3/1/001 16510937 PMC2583360

[B15] Cacheaux L. P. Ivens S. David Y. Lakhter A. J. Bar-Klein G. Shapira M. (2009). Transcriptome profiling reveals TGF-beta signaling involvement in epileptogenesis. *J. Neurosci.* 29 8927–8935. 10.1523/JNEUROSCI.0430-09.2009 19605630 PMC2875073

[B16] Campos A. C. P. Kikuchi D. S. Paschoa A. F. N. Kuroki M. A. Fonoff E. T. Hamani C. (2020). Unraveling the Role of Astrocytes in Subthalamic Nucleus Deep Brain Stimulation in a Parkinson’s Disease Rat Model. *Cell Mol. Neurobiol.* 40 939–954. 10.1007/s10571-019-00784-3 31939008 PMC7295825

[B17] Cancel L. M. Arias K. Bikson M. Tarbell J. M. (2018). Direct current stimulation of endothelial monolayers induces a transient and reversible increase in transport due to the electroosmotic effect. *Sci. Rep.* 8:9265. 10.1038/s41598-018-27524-9 29915178 PMC6006334

[B18] Cedeño D. L. Vallejo R. Kelley C. A. Platt D. C. Litvak L. M. Straka M. (2021). Modulation of Glia-Mediated Processes by Spinal Cord Stimulation in Animal Models of Neuropathic Pain. *Front. Pain Res.* 2:702906. 10.3389/fpain.2021.702906 35295479 PMC8915735

[B19] Cha M. Lee K. H. Lee B. H. (2020). Astroglial changes in the zona incerta in response to motor cortex stimulation in a rat model of chronic neuropathy. *Sci. Rep.* 10:943. 10.1038/s41598-020-57797-y 31969638 PMC6976635

[B20] Chen C. Bai X. Ding Y. Lee I. S. (2019). Electrical stimulation as a novel tool for regulating cell behavior in tissue engineering. *Biomater. Res.* 23:25. 10.1186/s40824-019-0176-8 31844552 PMC6896676

[B21] Chen K. Stieger K. C. Kozai T. D. (2021). Challenges and opportunities of advanced gliomodulation technologies for excitation-inhibition balance of brain networks. *Curr. Opin. Biotechnol.* 72 112–120. 10.1016/j.copbio.2021.10.008 34773740 PMC8671375

[B22] Chen Y. C. Zhu G. Y. Wang X. Shi L. Du T. T. Liu D. F. (2017). Anterior thalamic nuclei deep brain stimulation reduces disruption of the blood-brain barrier, albumin extravasation, inflammation and apoptosis in kainic acid-induced epileptic rats. *Neurol. Res.* 39 1103–1113. 10.1080/01616412.2017.1379241 28918702

[B23] Chung H. Im C. Seo H. Jun S. C. (2020). Morphological Influence and Electric Field Direction’s Influence on Activation of Cortical Neurons in Electrical Brain Stimulation: A Computational Study. *Annu. Int. Conf. IEEE Eng. Med. Biol. Soc.* 2020 2938–2941. 10.1109/EMBC44109.2020.9175250 33018622

[B24] Clancy J. A. Mary D. A. Witte K. K. Greenwood J. P. Deuchars S. A. Deuchars J. (2014). Non-invasive vagus nerve stimulation in healthy humans reduces sympathetic nerve activity. *Brain Stimulation* 7 871–877. 10.1016/j.brs.2014.07.031 25164906

[B25] Cogan S. F. (2008). Neural stimulation and recording electrodes. *Annu. Rev. Biomed. Eng.* 10 275–309. 10.1146/annurev.bioeng.10.061807.160518 18429704

[B26] Cogan S. F. Ehrlich J. Plante T. D. Van Wagenen R. (2009). Penetrating microelectrode arrays with low-impedance sputtered iridium oxide electrode coatings. *Annu. Int. Conf. IEEE Eng. Med. Biol. Soc.* 2009 7147–7150. 10.1109/IEMBS.2009.5335359 19965266 PMC7441535

[B27] Collaborators G. U. N. D. Feigin V. L. Vos T. Alahdab F. Amit A. M. L. Bärnighausen T. W. (2021). Burden of Neurological Disorders Across the US From 1990-2017: A Global Burden of Disease Study. *JAMA Neurol.* 78 165–176. 10.1001/jamaneurol.2020.4152 33136137 PMC7607495

[B28] Couloigner V. Gratacap M. Ambert-Dahan E. Borel S. Ettienne V. Kerouedan A. (2014). [A report of three cases and review of auditory brainstem implants in children]. *Neurochirurgie* 60 17–26. 10.1016/j.neuchi.2014.01.002 24656883

[B29] DaSilva A. F. Volz M. S. Bikson M. Fregni F. (2011). Electrode positioning and montage in transcranial direct current stimulation**. *J. Vis. Exp.* 51:2744. 10.3791/2744 21654618 PMC3339846

[B30] De La Cruz P. Fama C. Roth S. Haller J. Wilock M. Lange S. (2015). Predictors of Spinal Cord Stimulation Success. *Neuromodulation* 18 599–602. 10.1111/ner.12325 26119040 PMC4615463

[B31] Delaville C. Deurwaerdere P. D. Benazzouz A. (2011). Noradrenaline and Parkinson’s disease. *Front. Syst. Neurosci.* 5:31. 10.3389/fnsys.2011.00031 21647359 PMC3103977

[B32] Denoyer Y. Merlet I. Wendling F. Benquet P. (2020). Modelling acute and lasting effects of tDCS on epileptic activity. *J. Comput. Neurosci.* 48 161–176. 10.1007/s10827-020-00745-6 32307640

[B33] Deuschl G. Schade-Brittinger C. Krack P. Volkmann J. Schäfer H. Bötzel K. (2006). A randomized trial of deep-brain stimulation for Parkinson’s disease. *N. Engl. J. Med.* 355 896–908. 10.1056/NEJMoa060281 16943402

[B34] Eles J. Vazquez A. Kozai T. Cui X. (2019). Meningeal inflammatory response and fibrous tissue remodeling around intracortical implants: An in vivo two-photon imaging study. *Biomaterials* 195 111–123. 10.1016/j.biomaterials.2018.12.031 30634095 PMC6350934

[B35] Eles J. R. Vazquez A. L. Kozai T. D. Cui X. T. (2018). In vivo imaging of neuronal calcium during electrode implantation: Spatial and temporal mapping of damage and recovery. *Biomaterials* 174 79–94. 10.1016/j.biomaterials.2018.04.043 29783119 PMC5987772

[B36] Ereifej E. S. Rial G. M. Hermann J. K. Smith C. S. Meade S. M. Rayyan J. M. (2018). Implantation of Neural Probes in the Brain Elicits Oxidative Stress. *Front. Bioeng Biotechnol.* 6:9. 10.3389/fbioe.2018.00009 29487848 PMC5816578

[B37] Erickson M. A. Banks W. A. (2018). Neuroimmune Axes of the Blood-Brain Barriers and Blood-Brain Interfaces: Bases for Physiological Regulation, Disease States, and Pharmacological Interventions. *Pharmacol. Rev.* 70 278–314. 10.1124/pr.117.014647 29496890 PMC5833009

[B38] Farkas E. Luiten P. G. M. (2001). Cerebral microvascular pathology in aging and Alzheimer’s disease. *Progress Neurobiol.* 64 575–611. 10.1016/S0301-0082(00)00068-X 11311463

[B39] Feigin V. L. Abajobir A. A. Abate K. H. Abd-Allah F. Abdulle A. M. Abera S. F. (2017). Global, regional, and national burden of neurological disorders during 1990–2015: A systematic analysis for the Global Burden of Disease Study 2015. *Lancet Neurol.* 16 877–897. 10.1016/S1474-4422(17)30299-5 28931491 PMC5641502

[B40] Fenoy A. J. Goetz L. Chabardes S. Xia Y. (2014). Deep brain stimulation: Are astrocytes a key driver behind the scene? *CNS Neurosci. Ther.* 20 191–201. 10.1111/cns.12223 24456263 PMC3969941

[B41] Fernández E. Alfaro A. Soto-Sánchez C. Gonzalez-Lopez P. Lozano A. M. Peña S. (2021). Visual percepts evoked with an intracortical 96-channel microelectrode array inserted in human occipital cortex. *J. Clin. Investig.* 131:e151331. 10.1172/JCI151331 34665780 PMC8631600

[B42] Fifer D. P. M. M. S. Thomas T. M. Osborn L. E. Nickl R. W. Candrea D. N. Pohlmeyer E. A. (2020). Intracortical Microstimulation Elicits Human Fingertip Sensations**. *medRxiv.* [preprind]. 10.1101/2020.05.29.20117374

[B43] Flesher S. N. Collinger J. L. Foldes S. T. Weiss J. M. Downey J. E. Tyler-Kabara E. C. (2016). Intracortical microstimulation of human somatosensory cortex. *Sci. Transl. Med.* 8:361ra141. 10.1126/scitranslmed.aaf8083 27738096

[B44] Flesher S. N. Downey J. E. Weiss J. M. Hughes C. L. Herrera A. J. Tyler-Kabara E. C. (2021). A brain-computer interface that evokes tactile sensations improves robotic arm control. *Science* 372 831–836. 10.1126/science.abd0380 34016775 PMC8715714

[B45] Florence G. Dahlem M. A. Almeida A. C. Bassani J. W. Kurths J. (2009). The role of extracellular potassium dynamics in the different stages of ictal bursting and spreading depression: A computational study. *J. Theor. Biol.* 258 219–228. 10.1016/j.jtbi.2009.01.032 19490858

[B46] Foroushani A. N. Pack C. C. Sawan M. (2018). Cortical visual prostheses: From microstimulation to functional percept. *J. Neural. Eng.* 15:021005. 10.1088/1741-2552/aaa904 29350199

[B47] Forst J. C. Blok D. C. Slopsema J. P. Boss J. M. Heyboer L. A. Tobias C. M. (2015). Surface electrical stimulation to evoke referred sensation. *J. Rehabil. Res. Dev.* 52 397–406. 10.1682/JRRD.2014.05.0128 26348194

[B48] Fregni F. Boggio P. S. Mansur C. G. Wagner T. Ferreira M. J. Lima M. C. (2005). Transcranial direct current stimulation of the unaffected hemisphere in stroke patients. *Neuroreport* 16 1551–1555. 10.1097/01.wnr.0000177010.44602.5e 16148743

[B49] Gandiga P. C. Hummel F. C. Cohen L. G. (2006). Transcranial DC stimulation (tDCS): A tool for double-blind sham-controlled clinical studies in brain stimulation. *Clin. Neurophysiol.* 117 845–850. 10.1016/j.clinph.2005.12.003 16427357

[B50] Gellner A. K. Reis J. Fiebich B. L. Fritsch B. (2021). Electrified microglia: Impact of direct current stimulation on diverse properties of the most versatile brain cell. *Brain Stimul.* 14 1248–1258. 10.1016/j.brs.2021.08.007 34411753

[B51] Gellner A. K. Reis J. Fritsch B. (2016). Glia: A Neglected Player in Non-invasive Direct Current Brain Stimulation. *Front. Cell Neurosci.* 10:188. 10.3389/fncel.2016.00188 27551261 PMC4976108

[B52] Gellner A. K. Reis J. Holtick C. Schubert C. Fritsch B. (2020). Direct current stimulation-induced synaptic plasticity in the sensorimotor cortex: Structure follows function. *Brain Stimul.* 13 80–88. 10.1016/j.brs.2019.07.026 31405790

[B53] Gimenes C. Malheiros J. M. Battapady H. Tannus A. Hamani C. Covolan L. (2019). The neural response to deep brain stimulation of the anterior nucleus of the thalamus: A MEMRI and c-Fos study. *Brain Res. Bull.* 147 133–139. 10.1016/j.brainresbull.2019.01.011 30658130

[B54] Gondard E. Chau H. N. Mann A. Tierney T. S. Hamani C. Kalia S. K. (2015). Rapid Modulation of Protein Expression in the Rat Hippocampus Following Deep Brain Stimulation of the Fornix. *Brain Stimul.* 8 1058–1064. 10.1016/j.brs.2015.07.044 26321354

[B55] Gooch C. L. Pracht E. Borenstein A. R. (2017). The burden of neurological disease in the United States: A summary report and call to action. *Ann. Neurol.* 81 479–484. 10.1002/ana.24897 28198092

[B56] Hamani C. Temel Y. (2012). Deep brain stimulation for psychiatric disease: Contributions and validity of animal models. *Sci. Transl. Med.* 4:142rv8. 10.1126/scitranslmed.3003722 22786683

[B57] He B. Lu Z. He W. Huang B. Jiang H. (2016). Autonomic Modulation by Electrical Stimulation of the Parasympathetic Nervous System: An Emerging Intervention for Cardiovascular Diseases. *Cardiovasc. Ther.* 34 167–171. 10.1111/1755-5922.12179 26914959

[B58] Henning J. Koczan D. Glass A. Karopka T. Pahnke J. Rolfs A. (2007). Deep brain stimulation in a rat model modulates TH, CaMKIIa and Homer1 gene expression. *Eur. J. Neurosci.* 25 239–250. 10.1111/j.1460-9568.2006.05264.x 17241285

[B59] Herrington T. M. Cheng J. J. Eskandar E. N. (2016). Mechanisms of deep brain stimulation. *J. Neurophysiol.* 115 19–38. 10.1152/jn.00281.2015 26510756 PMC4760496

[B60] Howard-Quijano K. Yamaguchi T. Gao F. Kuwabara Y. Puig S. Lundquist E. (2021). Spinal cord stimulation reduces ventricular arrhythmias by attenuating reactive gliosis and activation of spinal interneurons. *Clin. Electrophysiol.* 7 1211–1225. 10.1016/j.jacep.2021.05.016 34454884 PMC8542625

[B61] Howland R. H. (2014). Vagus nerve stimulation. *Curr. Behav. Neurosci. Rep.* 1 64–73. 10.1007/s40473-014-0010-5 24834378 PMC4017164

[B62] Im J. J. Jeong H. Bikson M. Woods A. J. Unal G. Oh J. K. (2019). Effects of 6-month at-home transcranial direct current stimulation on cognition and cerebral glucose metabolism in Alzheimer’s disease. *Brain Stimul.* 12 1222–1228. 10.1016/j.brs.2019.06.003 31196835 PMC6703942

[B63] Ito D. Tanaka K. Suzuki S. Dembo T. Fukuuchi Y. (2001). Enhanced expression of Iba1, ionized calcium-binding adapter molecule 1, after transient focal cerebral ischemia in rat brain. *Stroke* 32 1208–1215. 10.1161/01.STR.32.5.1208 11340235

[B64] Jakobs M. Fomenko A. Lozano A. M. Kiening K. L. (2019). Cellular, molecular, and clinical mechanisms of action of deep brain stimulation-a systematic review on established indications and outlook on future developments. *EMBO Mol. Med.* 11:e9575. 10.15252/emmm.201809575 30862663 PMC6460356

[B65] Jang J. Kim H. Song Y. M. Park J.-U. (2019). Implantation of electronic visual prosthesis for blindness restoration**. *Optic. Materials Exp.* 9 3878–3894. 10.1364/OME.9.003878

[B66] Jobst B. C. Kapur R. Barkley G. L. Bazil C. W. Berg M. J. Bergey G. K. (2017). Brain-responsive neurostimulation in patients with medically intractable seizures arising from eloquent and other neocortical areas. *Epilepsia* 58 1005–1014. 10.1111/epi.13739 28387951

[B67] Kapural L. Yu C. Doust M. W. Gliner B. E. Vallejo R. Sitzman B. T. (2015). Novel 10-kHz High-frequency Therapy (HF10 Therapy) Is Superior to Traditional Low-frequency Spinal Cord Stimulation for the Treatment of Chronic Back and Leg Pain: The SENZA-RCT Randomized Controlled Trial. *Anesthesiology* 123 851–860. 10.1097/ALN.0000000000000774 26218762

[B68] Kettenmann H. Hanisch U.-K. Noda M. Verkhratsky A. (2011). Physiology of microglia. *Physiol. Rev.* 91 461–553. 10.1152/physrev.00011.2010 21527731

[B69] Khedr E. M. Salama R. H. Abdel Hameed M. Abo Elfetoh N. Seif P. (2019). Therapeutic Role of Transcranial Direct Current Stimulation in Alzheimer Disease Patients: Double-Blind, Placebo-Controlled Clinical Trial. *Neurorehabil. Neural. Repair* 33 384–394. 10.1177/1545968319840285 30940012

[B70] Kim J. W. Hwang J. H. Kim I. K. Kim Y. E. Yang H. J. Ehm G. (2013). Acute brain reaction to DBS electrodes after deep brain stimulation: Chronological observation. *Acta Neurochir.* 155 2365–2371. 10.1007/s00701-013-1853-3 24009047

[B71] Kim M. S. Koo H. Han S. W. Paulus W. Nitsche M. A. Kim Y. H. (2017). Repeated anodal transcranial direct current stimulation induces neural plasticity-associated gene expression in the rat cortex and hippocampus. *Restor. Neurol. Neurosci.* 35 137–146. 10.3233/RNN-160689 28059801

[B72] Korai S. A. Ranieri F. Di Lazzaro V. Papa M. Cirillo G. (2021). Neurobiological After-Effects of Low Intensity Transcranial Electric Stimulation of the Human Nervous System: From Basic Mechanisms to Metaplasticity. *Front. Neurol.* 12:587771. 10.3389/fneur.2021.587771 33658972 PMC7917202

[B73] Kozai T. D. Eles J. R. Vazquez A. L. Cui X. T. (2016). Two-photon imaging of chronically implanted neural electrodes: Sealing methods and new insights. *J. Neurosci. Methods* 258 46–55. 10.1016/j.jneumeth.2015.10.007 26526459 PMC4771525

[B74] Kozai T. D. Vazquez A. L. Weaver C. L. Kim S. G. Cui X. T. (2012). In vivo two-photon microscopy reveals immediate microglial reaction to implantation of microelectrode through extension of processes. *J. Neural. Eng.* 9:066001. 10.1088/1741-2560/9/6/066001 PMC3511663 23075490

[B75] Kozai T. D. Y. Jaquins-Gerstl A. S. Vazquez A. L. Michael A. C. Cui X. T. (2015). Brain Tissue Responses to Neural Implants Impact Signal Sensitivity and Intervention Strategies. *ACS Chemical. Neurosci.* 6 48–67. 10.1021/cn500256e 25546652 PMC4304489

[B76] Kronberg G. Rahman A. Sharma M. Bikson M. Parra L. C. (2020). Direct current stimulation boosts hebbian plasticity in vitro. *Brain Stimul.* 13 287–301. 10.1016/j.brs.2019.10.014 31668982 PMC6989352

[B77] Kuncel A. M. Grill W. M. (2004). Selection of stimulus parameters for deep brain stimulation. *Clin. Neurophysiol.* 115 2431–2441. 10.1016/j.clinph.2004.05.031 15465430

[B78] Lee H. U. Blasiak A. Agrawal D. R. Loong D. T. B. Thakor N. V. All A. H. (2017). Subcellular electrical stimulation of neurons enhances the myelination of axons by oligodendrocytes. *PLoS One* 12:e0179642. 10.1371/journal.pone.0179642 28671962 PMC5495216

[B79] Liu A. Vöröslakos M. Kronberg G. Henin S. Krause M. R. Huang Y. (2018). Immediate neurophysiological effects of transcranial electrical stimulation. *Nat. commun.* 9:5092. 10.1038/s41467-018-07233-7 30504921 PMC6269428

[B80] Lozano A. M. Lipsman N. (2013). Probing and regulating dysfunctional circuits using deep brain stimulation. *Neuron* 77 406–424. 10.1016/j.neuron.2013.01.020 23395370

[B81] Lozano A. M. Lipsman N. Bergman H. Brown P. Chabardes S. Chang J. W. (2019). Deep brain stimulation: Current challenges and future directions. *Nat. Rev. Neurol.* 15 148–160. 10.1038/s41582-018-0128-2 30683913 PMC6397644

[B82] Maisiyiti A. Chen J. D. (2019). Systematic review on gastric electrical stimulation in obesity treatment. *Exp. Rev. Med. Devices* 16 855–861. 10.1080/17434440.2019.1673728 31570014 PMC6946629

[B83] Malaga K. A. Schroeder K. E. Patel P. R. Irwin Z. T. Thompson D. E. Nicole Bentley J. (2015). Data-driven model comparing the effects of glial scarring and interface interactions on chronic neural recordings in non-human primates. *J. Neural. Eng.* 13:016010. 10.1088/1741-2560/13/1/016010 26655972

[B84] Matias C. M. Sharan A. Wu C. (2019). Responsive Neurostimulation for the Treatment of Epilepsy. *Neurosurg. Clin. N. Am.* 30 231–242. 10.1016/j.nec.2018.12.006 30898274

[B85] Mayberg H. S. Lozano A. M. Voon V. McNeely H. E. Seminowicz D. Hamani C. (2005). Deep brain stimulation for treatment-resistant depression. *Neuron* 45 651–660. 10.1016/j.neuron.2005.02.014 15748841

[B86] McClure D. Greenman S. C. Koppolu S. S. Varvara M. Yaseen Z. S. Galynker I. I. (2015). A Pilot Study of Safety and Efficacy of Cranial Electrotherapy Stimulation in Treatment of Bipolar II Depression. *J. Nerv. Ment. Dis.* 203 827–835. 10.1097/NMD.0000000000000378 26414234 PMC4892785

[B87] McCreery D. B. Agnew W. F. Yuen T. G. H. Bullara L. (1990). Charge density and charge per phase as cofactors in neural injury induced by electrical stimulation. *IEEE Trans. Biomed. Eng.* 37 996–1001. 10.1109/10.102812 2249872

[B88] McIntyre C. C. Anderson R. W. (2016). Deep brain stimulation mechanisms: The control of network activity via neurochemistry modulation. *J. Neurochem.* 139 338–345. 10.1111/jnc.13649 27273305 PMC5358920

[B89] Merrill D. R. Bikson M. Jefferys J. G. (2005). Electrical stimulation of excitable tissue: Design of efficacious and safe protocols. *J. Neurosci. Methods* 141 171–198. 10.1016/j.jneumeth.2004.10.020 15661300

[B90] Micera S. Keller T. Lawrence M. Morari M. Popovic D. B. (2010). Wearable neural prostheses. Restoration of sensory-motor function by transcutaneous electrical stimulation. *IEEE Eng. Med. Biol. Mag.* 29 64–69. 10.1109/MEMB.2010.936547 20659859

[B91] Michelson N. J. Vazquez A. L. Eles J. R. Salatino J. W. Purcell E. K. Williams J. J. (2018). Multi-scale, multi-modal analysis uncovers complex relationship at the brain tissue-implant neural interface: New emphasis on the biological interface. *J. Neural. Eng.* 15:033001. 10.1088/1741-2552/aa9dae 29182149 PMC5967409

[B92] Milighetti S. Sterzi S. Fregni F. Hanlon C. A. Hayley P. Murphy M. D. (2020). Effects of tDCS on spontaneous spike activity in a healthy ambulatory rat model. *Brain Stimul.* 13 1566–1576. 10.1016/j.brs.2020.08.016 32927094 PMC7722157

[B93] Miocinovic S. Somayajula S. Chitnis S. Vitek J. L. (2013). History, applications, and mechanisms of deep brain stimulation. *JAMA Neurol.* 70 163–171. 10.1001/2013.jamaneurol.45 23407652

[B94] Miron V. E. Boyd A. Zhao J. W. Yuen T. J. Ruckh J. M. Shadrach J. L. (2013). M2 microglia and macrophages drive oligodendrocyte differentiation during CNS remyelination. *Nat. Neurosci.* 16 1211–1218. 10.1038/nn.3469 23872599 PMC3977045

[B95] Mishima T. Nagai T. Yahagi K. Akther S. Oe Y. Monai H. (2019). Transcranial Direct Current Stimulation (tDCS) Induces Adrenergic Receptor-Dependent Microglial Morphological Changes in Mice. *eNeuro* 6:ENEURO.204–ENEURO.219. 10.1523/ENEURO.0204-19.2019 31444225 PMC6751370

[B96] Monai H. Hirase H. (2018). Astrocytes as a target of transcranial direct current stimulation (tDCS) to treat depression. *Neurosci. Res.* 126 15–21. 10.1016/j.neures.2017.08.012 29079367

[B97] Monai H. Ohkura M. Tanaka M. Oe Y. Konno A. Hirai H. (2016). Calcium imaging reveals glial involvement in transcranial direct current stimulation-induced plasticity in mouse brain. *Nat. Commun.* 7:11100. 10.1038/ncomms11100 27000523 PMC4804173

[B98] Murphey D. K. Maunsell J. H. Beauchamp M. S. Yoshor D. (2009). Perceiving electrical stimulation of identified human visual areas. *Proc. Natl. Acad. Sci.* 106 5389–5393. 10.1073/pnas.0804998106 19276119 PMC2664020

[B99] Nagy B. Hovhannisyan A. Barzan R. Chen T. J. Kukley M. (2017). Different patterns of neuronal activity trigger distinct responses of oligodendrocyte precursor cells in the corpus callosum. *PLoS Biol.* 15:e2001993. 10.1371/journal.pbio.2001993 28829781 PMC5567905

[B100] Nair D. R. Laxer K. D. Weber P. B. Murro A. M. Park Y. D. Barkley G. L. (2020). Nine-year prospective efficacy and safety of brain-responsive neurostimulation for focal epilepsy. *Neurology* 95:e1244–e1256. 10.1212/WNL.0000000000010154 32690786 PMC7538230

[B101] Nazzaro J. M. Lyons K. E. Pahwa R. (2013). Deep brain stimulation for essential tremor. *Handb. Clin. Neurol.* 116 155–166. 10.1016/B978-0-444-53497-2.00013-9 24112892

[B102] Nimmerjahn A. Kirchhoff F. Helmchen F. (2005). Resting microglial cells are highly dynamic surveillants of brain parenchyma in vivo. *Science* 308 1314–1318. 10.1126/science.1110647 15831717

[B103] Nitsche M. A. Cohen L. G. Wassermann E. M. Priori A. Lang N. Antal A. (2008). Transcranial direct current stimulation: State of the art 2008. *Brain Stimul.* 1 206–223. 10.1016/j.brs.2008.06.004 20633386

[B104] Nitsche M. A. Paulus W. (2001). Sustained excitability elevations induced by transcranial DC motor cortex stimulation in humans. *Neurology* 57 1899–1901. 10.1212/WNL.57.10.1899 11723286

[B105] Oakley J. C. Espinosa F. Bothe H. McKean J. Allen P. Burchiel K. (2006). Transverse Tripolar Spinal Cord Stimulation: Results of an International Multicenter Study. *Neuromodulation* 9 192–203. 10.1111/j.1525-1403.2006.00060.x 22151707

[B106] O’Connell N. E. Marston L. Spencer S. DeSouza L. H. Wand B. M. (2018). Non-invasive brain stimulation techniques for chronic pain. *Cochrane Database Syst. Rev.* 4:CD008208. 10.1002/14651858.CD008208.pub4 29652088 PMC6494527

[B107] Pancrazio J. J. Deku F. Ghazavi A. Stiller A. M. Rihani R. Frewin C. L. (2017). Thinking Small: Progress on Microscale Neurostimulation Technology. *Neuromodulation* 20 745–752. 10.1111/ner.12716 29076214 PMC5943060

[B108] Parker R. A. Davis T. S. House P. A. Normann R. A. Greger B. (2011). The functional consequences of chronic, physiologically effective intracortical microstimulation. *Prog. Brain Res.* 194 145–165. 10.1016/B978-0-444-53815-4.00010-8 21867801 PMC7709570

[B109] Parthasarathy H. B. Graybiel A. M. (1997). Cortically driven immediate-early gene expression reflects modular influence of sensorimotor cortex on identified striatal neurons in the squirrel monkey. *J. Neurosci.* 17 2477–2491. 10.1523/JNEUROSCI.17-07-02477.1997 9065508 PMC6573482

[B110] Pelletier S. J. Cicchetti F. (2014). Cellular and molecular mechanisms of action of transcranial direct current stimulation: Evidence from in vitro and in vivo models. *Int. J. Neuropsychopharmacol.* 18:yu047. 10.1093/ijnp/pyu047 25522391 PMC4368894

[B111] Pelletier S. J. Lagace M. St-Amour I. Arsenault D. Cisbani G. Chabrat A. (2014). The morphological and molecular changes of brain cells exposed to direct current electric field stimulation. *Int. J. Neuropsychopharmacol.* 18:yu090. 10.1093/ijnp/pyu090 25522422 PMC4376545

[B112] Peruzzotti-Jametti L. Cambiaghi M. Bacigaluppi M. Gallizioli M. Gaude E. Mari S. (2013). Safety and efficacy of transcranial direct current stimulation in acute experimental ischemic stroke. *Stroke* 44 3166–3174. 10.1161/STROKEAHA.113.001687 23982710

[B113] Pienaar I. S. Lee C. H. Elson J. L. McGuinness L. Gentleman S. M. Kalaria R. N. (2015). Deep-brain stimulation associates with improved microvascular integrity in the subthalamic nucleus in Parkinson’s disease. *Neurobiol. Dis.* 74 392–405. 10.1016/j.nbd.2014.12.006 25533682

[B114] Pikhovych A. Stolberg N. P. Jessica Flitsch L. Walter H. L. Graf R. Fink G. R. (2016). Transcranial Direct Current Stimulation Modulates Neurogenesis and Microglia Activation in the Mouse Brain. *Stem. Cells Int.* 2016:2715196. 10.1155/2016/2715196 27403166 PMC4925996

[B115] Pudenz R. H. Bullara L. Dru D. Talalla A. (1975). Electrical stimulation of the brain. II. Effects on the blood-brain barrier. *Surgical. Neurol.* 4 265–270. 1162603

[B116] Rajan A. T. Boback J. L. Dammann J. F. Tenore F. V. Wester B. A. Otto K. J. (2015). The effects of chronic intracortical microstimulation on neural tissue and fine motor behavior. *J. Neural. Eng.* 12:066018. 10.1088/1741-2560/12/6/066018 PMC8129590 26479701

[B117] Ralay Ranaivo H. Wainwright M. S. (2010). Albumin activates astrocytes and microglia through mitogen-activated protein kinase pathways. *Brain Res.* 1313 222–231. 10.1016/j.brainres.2009.11.063 19961838 PMC2812578

[B118] Ranck J. B. Jr. (1975). Which elements are excited in electrical stimulation of mammalian central nervous system: A review. *Brain Res.* 98 417–440. 10.1016/0006-8993(75)90364-9 1102064

[B119] Rigoard P. Billot M. Ingrand P. Durand-Zaleski I. Roulaud M. Peruzzi P. (2021). How Should we Use Multicolumn Spinal Cord Stimulation to Optimize Back Pain Spatial Neural Targeting? A Prospective, Multicenter, Randomized, Double-Blind, Controlled Trial (ESTIMET Study). *Neuromodulation* 24 86–101. 10.1111/ner.13251 32865344

[B120] Robin L. M. Oliveira da Cruz J. F. Langlais V. C. Martin-Fernandez M. Metna-Laurent M. Busquets-Garcia A. (2018). Astroglial CB1 Receptors Determine Synaptic D-Serine Availability to Enable Recognition Memory. *Neuron* 98:935–944e5. 10.1016/j.neuron.2018.04.034 29779943

[B121] Rueger M. A. Keuters M. H. Walberer M. Braun R. Klein R. Sparing R. (2012). Multi-session transcranial direct current stimulation (tDCS) elicits inflammatory and regenerative processes in the rat brain. *PLoS One* 7:e43776. 10.1371/journal.pone.0043776 22928032 PMC3425495

[B122] Salas M. A. Bashford L. Kellis S. Jafari M. Jo H. Kramer D. (2018). Proprioceptive and cutaneous sensations in humans elicited by intracortical microstimulation. *eLife* 7:e32904. 10.7554/eLife.32904 29633714 PMC5896877

[B123] Salatino J. W. Ludwig K. A. Kozai T. D. Y. Purcell E. K. (2017). Glial responses to implanted electrodes in the brain. *Nat. Biomed. Eng.* 1 862–877. 10.1038/s41551-017-0154-1 30505625 PMC6261524

[B124] Sarmento A. Borges N. Lima D. (1994). Influence of electrical stimulation of locus coeruleus on the rat blood-brain barrier permeability to sodium fluorescein. *Acta Neurochirurgica* 127 215–219. 10.1007/BF01808769 7942206

[B125] Saxena T. Karumbaiah L. Gaupp E. A. Patkar R. Patil K. Betancur M. (2013). The impact of chronic blood-brain barrier breach on intracortical electrode function. *Biomaterials* 34 4703–4713. 10.1016/j.biomaterials.2013.03.007 23562053

[B126] Schiefer M. Tan D. Sidek S. M. Tyler D. J. (2016). Sensory feedback by peripheral nerve stimulation improves task performance in individuals with upper limb loss using a myoelectric prosthesis. *J. Neural. Eng.* 13:016001. 10.1088/1741-2560/13/1/016001 PMC5517302 26643802

[B127] Sehic A. Guo S. Cho K. S. Corraya R. M. Chen D. F. Utheim T. P. (2016). Electrical Stimulation as a Means for Improving Vision. *Am. J. Pathol.* 186 2783–2797. 10.1016/j.ajpath.2016.07.017 27643530 PMC5225285

[B128] Shine A. Abell T. L. (2020). Role of gastric electrical stimulation in the treatment of gastroparesis. *Gastrointestinal. Disord.* 2 12–26. 10.3390/gidisord2010003

[B129] Sofroniew M. V. Vinters H. V. (2010). Astrocytes: Biology and pathology. *Acta Neuropathologica* 119 7–35. 10.1007/s00401-009-0619-8 20012068 PMC2799634

[B130] Solomons C. D. Shanmugasundaram V. (2020). Transcranial direct current stimulation: A review of electrode characteristics and materials. *Med. Eng. Phys.* 85 63–74. 10.1016/j.medengphy.2020.09.015 33081965

[B131] Theodore W. H. Fisher R. (2007). Brain stimulation for epilepsy. *Acta Neurochir. Suppl.* 97 261–272. 10.1007/978-3-211-33081-4_29 17691312

[B132] Uc E. Y. Follett K. A. (2007). Deep brain stimulation in movement disorders. *Semin. Neurol.* 27 170–182. 10.1055/s-2007-971175 17390262

[B133] Vallejo R. Gupta A. Kelley C. A. Vallejo A. Rink J. Williams J. M. (2020). Effects of phase polarity and charge balance spinal cord stimulation on behavior and gene expression in a rat model of neuropathic pain. *Neuromodulation* 23 26–35. 10.1111/ner.12964 31070863

[B134] van Vliet E. A. da S. Costa Araujo Redeker S. van Schaik R. Aronica E. (2007). Blood-brain barrier leakage may lead to progression of temporal lobe epilepsy. *Brain* 130 521–534. 10.1093/brain/awl318 17124188

[B135] Varoli E. Pisoni A. Mattavelli G. C. Vergallito A. Gallucci A. Mauro L. D. (2018). Tracking the Effect of Cathodal Transcranial Direct Current Stimulation on Cortical Excitability and Connectivity by Means of TMS-EEG. *Front. Neurosci.* 12:319. 10.3389/fnins.2018.00319 29867330 PMC5962888

[B136] Vedam-Mai V. Baradaran-Shoraka M. Reynolds B. A. Okun M. S. (2016). Tissue Response to Deep Brain Stimulation and Microlesion: A Comparative Study. *Neuromodulation* 19 451–458. 10.1111/ner.12406 27018335 PMC4961567

[B137] Vedam-Mai V. Krock N. Ullman M. Foote K. D. Shain W. Smith K. (2011). The national DBS brain tissue network pilot study: Need for more tissue and more standardization. *Cell Tissue Banking* 12 219–231. 10.1007/s10561-010-9189-1 20589432

[B138] Vedam-Mai V. van Battum E. Y. Kamphuis W. Feenstra M. G. Denys D. Reynolds B. A. (2012). Deep brain stimulation and the role of astrocytes. *Mol. Psychiatry* 17 124–131. 10.1038/mp.2011.61 21625231

[B139] Wark H. Sharma R. Mathews K. Fernandez E. Yoo J. Christensen B. (2013). A new high-density (25 electrodes/mm2) penetrating microelectrode array for recording and stimulating sub-millimeter neuroanatomical structures. *J. Neural. Eng.* 10:045003. 10.1088/1741-2560/10/4/045003 23723133

[B140] Wellman S. M. Cambi F. Kozai T. D. (2018). The role of oligodendrocytes and their progenitors on neural interface technology: A novel perspective on tissue regeneration and repair. *Biomaterials* 183 200–217. 10.1016/j.biomaterials.2018.08.046 30172245 PMC6469877

[B141] Wilson B. S. Dorman M. F. (2008). Cochlear implants: A remarkable past and a brilliant future. *Hear. Res.* 242 3–21. 10.1016/j.heares.2008.06.005 18616994 PMC3707130

[B142] Woeppel K. Yang Q. Cui X. T. (2017). Recent Advances in Neural Electrode-Tissue Interfaces. *Curr. Opin. Biomed. Eng.* 4 21–31. 10.1016/j.cobme.2017.09.003 29423457 PMC5798641

[B143] Wolburg H. Noell S. Mack A. Wolburg-Buchholz K. Fallier-Becker P. (2009). Brain endothelial cells and the glio-vascular complex. *Cell Tissue Res.* 335 75–96. 10.1007/s00441-008-0658-9 18633647

[B144] Wu H. Hariz M. Visser-Vandewalle V. Zrinzo L. Coenen V. A. Sheth S. A. (2021). Deep brain stimulation for refractory obsessive-compulsive disorder (OCD): Emerging or established therapy? *Mol. Psychiatry* 26 60–65. 10.1038/s41380-020-00933-x 33144712 PMC7815503

[B145] Wu Y. C. Liao Y. S. Yeh W. H. Liang S. F. Shaw F. Z. (2021). Directions of Deep Brain Stimulation for Epilepsy and Parkinson’s Disease. *Front. Neurosci.* 15:680938. 10.3389/fnins.2021.680938 34194295 PMC8236576

[B146] Xu J. Zheng X. Cheng K. K. Chang X. Shen G. Liu M. (2017). NMR-based metabolomics Reveals Alterations of Electro-acupuncture Stimulations on Chronic Atrophic Gastritis Rats. *Sci. Rep.* 7:45580. 10.1038/srep45580 28358020 PMC5372362

[B147] Yang A. I. Buch V. P. Heman-Ackah S. M. Ramayya A. G. Hitti F. L. Beatson N. (2020). Thalamic Deep Brain Stimulation for Essential Tremor: Relation of the Dentatorubrothalamic Tract with Stimulation Parameters. *World Neurosurg* 137:e89–e97. 10.1016/j.wneu.2020.01.039 31954907 PMC7584387

[B148] Yang Q. Vazquez A. L. Cui X. T. (2021). Long-term in vivo two-photon imaging of the neuroinflammatory response to intracortical implants and micro-vessel disruptions in awake mice. *Biomaterials* 276:121060. 10.1016/j.biomaterials.2021.121060 34419839 PMC8409342

[B149] Yang Y. Yang L. Y. Orban L. Cuylear D. Thompson J. Simon B. (2018). Non-invasive vagus nerve stimulation reduces blood-brain barrier disruption in a rat model of ischemic stroke. *Brain Stimul.* 11 689–698. 10.1016/j.brs.2018.01.034 29496430 PMC6019567

[B150] Yap J. Y. Keatch C. Lambert E. Woods W. Stoddart P. R. Kameneva T. (2020). Critical review of transcutaneous vagus nerve stimulation: Challenges for translation to clinical practice. *Front. Neurosci.* 14:284. 10.3389/fnins.2020.00284 32410932 PMC7199464

[B151] Yue L. Falabella P. Christopher P. Wuyyuru V. Dorn J. Schor P. (2015). Ten-Year Follow-up of a Blind Patient Chronically Implanted with Epiretinal Prosthesis Argus I. *Ophthalmology* 122 2545–52e1. 10.1016/j.ophtha.2015.08.008 26386850

[B152] Yuru Tang R. W. Xiao Gao Dengbin A. (2018). New parameter adjustment with spinal cord stimulation for postherpetic neuralgia treatment: A case report and literature review. *Dermatologica Sinica* 36 232–235. 10.1016/j.dsi.2018.07.004

[B153] Zareen N. Dodson S. Armada K. Awad R. Sultana N. Hara E. (2018). Stimulation-dependent remodeling of the corticospinal tract requires reactivation of growth-promoting developmental signaling pathways. *Exp. Neurol.* 307 133–144. 10.1016/j.expneurol.2018.05.004 29729248 PMC6092758

[B154] Zeng F. G. Rebscher S. Harrison W. Sun X. Feng H. (2008). Cochlear implants: System design, integration, and evaluation. *IEEE Rev. Biomed. Eng.* 1 115–142. 10.1109/RBME.2008.2008250 19946565 PMC2782849

[B155] Zhao M. Bai H. Wang E. Forrester J. V. McCaig C. D. (2004). Electrical stimulation directly induces pre-angiogenic responses in vascular endothelial cells by signaling through VEGF receptors. *J. Cell Sci.* 117 397–405. 10.1242/jcs.00868 14679307 PMC1459284

[B156] Zheng X. S. Tan C. Castagnola E. Cui X. T. (2021a). Electrode Materials for Chronic Electrical Microstimulation. *Adv. Healthc. Mater.* 10:e2100119. 10.1002/adhm.202100119 34029008 PMC8257249

[B157] Zheng X. S. Yang Q. Vazquez A. L. Tracy Cui X. (2021b). Imaging the Efficiency of Poly(3,4-ethylenedioxythiophene) Doped with Acid-Functionalized Carbon Nanotube and Iridium Oxide Electrode Coatings for Microstimulation. *Adv. Nanobiomed. Res.* 1:2000092. 10.1002/anbr.202000092 34746928 PMC8552016

